# The role of artificial intelligence in hastening time to recruitment in clinical trials

**DOI:** 10.1259/bjro.20220023

**Published:** 2023-05-16

**Authors:** Abdalah Ismail, Talha Al-Zoubi, Issam El Naqa, Hina Saeed

**Affiliations:** 1 Advocate Aurora Health Care Department of Diagnostic Radiology, Aurora, United States; 2 MOFFITT Cancer Center, Tampa, United States; 3 Lynn Cancer Institute-Baptist Health City, Boca Raton, United States

## Abstract

Novel and developing artificial intelligence (AI) systems can be integrated into healthcare settings in numerous ways. For example, in the case of automated image classification and natural language processing, AI systems are beginning to demonstrate near expert level performance in detecting abnormalities such as seizure activity. This paper, however, focuses on AI integration into clinical trials. During the clinical trial recruitment process, considerable labor and time is spent sifting through electronic health record and interviewing patients. With the advancement of deep learning techniques such as natural language processing, intricate electronic health record data can be efficiently processed. This provides utility to workflows such as recruitment for clinical trials. Studies are starting to show promise in shortening the time to recruitment and reducing workload for those involved in clinical trial design. Additionally, numerous guidelines are being constructed to encourage integration of AI into the healthcare setting with meaningful impact. The goal would be to improve the clinical trial process by reducing bias in patient composition, improving retention of participants, and lowering costs and labor.

## INTRODUCTION

The success rate of clinical trials has been historically low. Overall, the probability of success for all drugs and vaccines is 13.8%.^1^ This figure catapults to 20.9% when excluding oncology drugs, which demonstrates a meager 3.4% success rate, while vaccines for infectious disease present a 33.4% success rate.^1^ There are many reasons which render clinical trials unsuccessful including: premature termination, failure to meet accrual goals and enrollment deadlines, and inefficiencies in the recruitment process leading to a mismatch between the patient and clinical study. However, the primary source of failure remains the inability of a clinical trial to demonstrate efficacy. Of course, there are many causes why a clinical study might fail to demonstrate efficacy such as: a flawed study design, unsuitable subjects, an inappropriate endpoint, or low power (small sample size).^2^ Feller reported that a quarter of all cancer trials actually failed to enroll a statistically powerful sample size and 18% of trials closed with less than half the target of enrollees.^3^ In a later study, Hwang et al assessed 640 Phase III trials with novel therapeutics and found that 54% failed in development of the clinical trial, with nearly 57% failing due to inadequate efficacy.^4^

On average, only 1 in 10 drugs entering a clinical trial reaches the market; this is after having spent a minimum of 10–15 years in research and development and 1.5–2 billion USD to bring that drug to fruition.^5^ Therefore, the expense of a failed clinical trial is shattering. This high failure rate is explained by the above barriers mentioned in our review, including recruiting techniques, cohort selection, as well as patient monitoring.

A number of solutions to the aforementioned issues have been proposed. To start with, a standardization of the recruitment process along with simplifying its implementation would allow for hastened recruitment and higher retention of participants.^6^ Additionally, engagement of other staff to co-ordinate enrollment, reduce time from ethics approval to first recruit, and serve as dedicated trial co-ordinators would shorten time to recruitment.^6^ However, the human element of emotion will ultimately prevent a perfectly standardized process. For instance, after assessing management of local post-surgical pain, the most important factor translating into positive recruitment was enthusiasm of the lead investigator.^2^ A quantitative measure is always in the running to assist with such failures.^7^ Through advancements in computing, quantitative measures are starting to gain greater traction due to artificial intelligence (AI) for application in clinical studies and clinical trials including patient accrual, monitoring, and retention.^6^

When designing a clinical trial, considerable effort is allocated towards recruiting the right patients.^8,9^ This involves eligibility assessments through interviews and physical exams, along with an extensive EMR review to reach decisions based on available patient data. Currently, studies exist that aim to make this a quicker and more reliable process by using AI to analyze large database samples. For instance, Rizzo et al emulated clinical trials by using a program called “Trial pathfinder,” an AI system, to demonstrate that relaxing eligibility criteria made clinical trials more inclusive.^10^ This would eliminate the need for complex paperwork, increased employees, and more clinic time to assess extensive eligibility criteria.^3^ Research is being conducted which uses AI systems to automate the recruitment process and reduce the time needed to develop an optimal patient composition.^11–13^ There have been companies leading the way in AI introduction into clinical trial design, including: Antidote, Deep 6 AI, Mendel.AI, IQVIA, and Watson for Clinical Trial Matching. For example, Mendel.AI is developing AI-intervention in massive data banks such as the Comprehensive Blood and Cancer Center to automate patient data for faster clinical trial recruitment processes.^14^ Our paper will focus on the role of AI to shorten time to recruitment in clinical trials, which remains an important factor in improving patient composition and fulfilling census for clinical research.

## Evolution of AI in clinical trials

Tracking back to the 1970s, AI systems were introduced to aid in diagnostics. The MYCIN Project was an early initiative at Stanford in the 1970s, which oversaw the development of a bacteria subtype identification system.^15^ Later, in the 1980s, computer-aided diagnosis became very popular as an adjunct or “second opinion” in diagnostic radiology. Moving on to the 90s and 2000s, these efforts continued but computing was simply too slow to implement it. Given the rapid progression in the field of AI, particularly in deep learning algorithms combined with enhancements in computing hardware, this technology is now becoming increasingly accessible.^16^ In one study exploring dermatologic-level classification of skin cancer, deep convolutional neural networks (CNNs) were used for the automated classification of skin lesions.^17^ It was shown that an AI image classification system achieved performance on par with all tested experts in identifying keratinocyte carcinomas *vs* benign seborrheic keratoses. Its competence was comparable to actual dermatologists.

Another example of AI in medicine is in radiation oncology. Typically, dose adjustments to reach the most effective target radiation are clinically subjective. For instance, a population of non-small cell lung cancer patients can have their treatment optimized by radiation dose adaptations using deep reinforcement learning (DRL).^18^ Positron emission tomography-fludeoxyglucose in certain treatments can be detected and adjusted mid-trial to escalate the radiation dose and improve local tumor control in these lung cancer patients. Using DRL, neural networks can use valuable patient data such as clinical and imaging features in a radiotherapy artificial environment (constructed by the network), to optimize dosing in a response-adapted treatment setting. This DRL achieved comparable decision-making than the clinical ones in terms of improving local control while minimizing toxicity when escalating radiation doses.^18^ A newer study using 6095 scalp EEGs from 2711 patients found that an AI system matched or exceeded experts in detecting seizure events.^11^ AI techniques have advanced to such a level of maturity that allows them to mimic real-life conditions and assist decision-making that is based on human subjective nature. These objective measures are much easier to interpret when it comes to discerning malignant *vs* benign tumors. For purposes of clinical trials, the concept becomes a lot more multifaceted. Despite objective measures widely available, there is simply not a sure way to guarantee that the masses of patient data will be accurately and efficiently interpreted to guide recruitment decisions in the best way. Thus, AI’s role is to attempt an idealization of the key steps of clinical trial design.

Another example is that of text mining of radiology reports using natural language processing (NLP). This annotates large data sets automatically which would reduce human error throughout this process.^19^ NLP is an interdisciplinary field at the boundary of linguistics, computer science, and AI. In essence, researchers in this area seek to take something as seemingly haphazard as human language and make it understandable by computers. In the context of healthcare, natural language data can refer to data that are found in documents, such as EMR. Presently, there exists Neural NLP which has more involvement with deep neural networks which help analyze notes and free text in EMR. The tasks that are performed by a given NLP system include search engines, speech recognition, and email spam filtering. Speech recognition involves a sound clip of a person speaking to determine the textual representation of the speech. In summary, these methods are meant to teach AI how to recognize nuances such as loose and free text and even images. This will expedite data analysis by saving time on labor of perusing through patient data.

## AI intervention to hasten time to recruitment

Deep learning algorithms ranging from CNN to recurrent neural networks can be applied to image classification and NLP respectively. Within the context of clinical trial recruitment, NLP can be used to label free EHR text into categories meant for screening clinical trial eligibility.^20^ These systems can handle large volumes of unstructured data, including many features. NLP can be used to automatically extract important information from clinical documentation, such as prior treatments, genetics, and diagnoses. This can save time for healthcare providers and reduce the risk of errors. This is highly advantageous in the context of healthcare because EHR is the perfect example of an unstructured data set. Depending on the application, these AI systems can be trained on EHR data sets to predict prognosis, length of hospital stay, and other outcomes. A study by Schevchenko et al showed that an AI trained system was able to predict the length of hospital stay based on analysis of EHR text data to an error of 2 days.^21^ Our focus will be on AI intervention to hasten time to recruitment in clinical trials and how these deep learning technologies accomplish this.

We can now break down the role of AI, and how it will enhance the clinical trial design process, into three metrics: patient recruitment, cohort composition, and patient monitoring. Once a patient is selected for a clinical trial, they may not be motivated to continue the trial. AI systems can aid in this by improving patient cohort composition through automatic eligibility assessments, simplification of trial designs, and automatic trial recommendations. In a study using pediatric oncology patients, it was found that using an AI system resulted in a 90% lower workload in the trial recruitment process.^22^ These automated processes not only optimize the cohort composition but are also much quicker in assembling the cohort itself which hastens time to recruitment. It is thought, *e.g.* in the Harrer paper, that trial designs and automated assessments and recommendations on a machine learned algorithm can quickly compile, recognize, and make decisions on large EMR data sets.^23^ This will circumvent the long and arduous recruitment process that involves employees manually extracting this data and having to interview patients. As for cohort composition, the ideal would be to use a multiomic profile, which represents a diverse genomic profile, proteomics, metabolomics, and metabolites to track drug therapy response. This multiomic profile combined with an EMR, imaging, and patient history/physical exam data is an immense volume of data to process. With this, EMRs can be consolidated into a uniform metric to assess a patient’s eligibility more easily into a certain composition. This would lead to faster decision-making based on patient data as it will be made uniform. Initially, a binary would be established as eligible or not eligible for the clinical trial. Based on a calculated probability, patients can be grouped into either being eligible or not eligible to partake in a clinical trial if they meet a predetermined probability threshold.

Recruiting is very labor intensive and takes considerable time to carry out. As discussed above, AI technologies can validate and recognize patient eligibility, thereby automating the screening process. One study published in JMIR medical informatics explores the effect of real-time automated patient screening systems for clinical trial eligibility in the emergency department.^24^ Data (both structured and unstructured) were used to train these algorithms to recognize patient suitability. Then, this system known as ACTES, was integrated into clinical practice to support real-time patient screening. The ACTES was fully integrated into the pediatric ED at Cincinnati children’s hospital and was successfully able to recommend potential candidates for clinical trials. Concurrently, a 12-month time-and-motion study as well as quantitative assessment of enrollment was performed to assess the system’s effectiveness. It was found that ACTES reduced patient screening time by 34%, by reducing time spent towards administrative tasks, conversations, and unstructured teamwork.^24^ On measures of effectiveness via system usability scales, the ACTES achieved 80%.

Additionally, an AI tool was used in a large community cancer center. It was shown, during a system-assisted eligibility determination, that the screening time for 90 patients took a fifth of the time as manual eligibility screening.^25^ Another study in Australian lung cancer patients showed that in 102 patients, an AI trial matching system screened patients in 15.5 s with a 91.6% accuracy for eligibility compared to the real-world assessment.^26^ These computerized solutions are proving very effective, and with further integration of AI systems into healthcare, the prolonged process of clinical trial recruitment that often causes insufficient power of studies due to low quantity of enrollees will be significantly reduced. However, there remains the issue of translating study results from hospital to hospital well known in the literature. Ultimately, there remains uncertainty that these AI implementations will yield meaningful healthcare outcomes.

Currently, a focus on trial eligibility criteria predominates. There is a computational framework called “Trial Pathfinder,” which uses Shapley values, which is the average expected marginal contribution of adding one criterion to the hazard ratio after all possible combinations of criteria are considered.^10^ This framework integrates real-world data from massive data banks and analyzes the hazard ratio of overall survival for cohorts defined by different eligibility criteria such as age, demographics, diagnosis date, and informed consent signature date. The idea is to expand eligibility by taking this individual data by applying the hazard ratio over different combinations of criteria to assess how it varies with larger patient cohorts. The concept is to relax eligibility criteria, so it is not overly restrictive and limits patients from beneficial treatments. Trial pathfinder was used to emulate completed trials of non-small cell lung cancer from nationwide databases with tens of thousands of patients. What was found was that many common criteria, including firm exclusions, had minimal effect on hazard ratios in these trials. When using this efficient, data-driven approach, the pool of eligible patients more than doubled and the hazard ratios increased a mere 0.05 on average.^10^ In machine learning, this has been proposed as an approach to make data more uniform and reliable. This suggests that time consuming, incredibly strict trial criteria using multiple screenings, interviews, and EMR reviews are certainly able to be more streamlined and automated without compromising the study design. A drawback to this study is that it uses retrospective data, which may not translate well clinically.

Moreover, a study by Whitty showed that AI systems, when applied to two completed oncology studies in breast and lung at the Comprehensive Blood and Cancer Center, resulted in a 24–50% increase in the number of patients identified as eligible compared to standard practices.^14^ Additionally, an average of 19 days in the case of breast cancer and 263 days in that of the lung elapsed between actual patient eligibility and identification when standard process was used. Of course, when using AI to automate eligibility, it has the potential to speed this process up considerably. The implication this has is obvious: a more effective and faster recruitment process will result in more patients due to its convenience and ability to precisely identify more eligible patients, especially in situations that standard processes would normally miss.

## DISCUSSION

The process of clinical trial recruitment is arduous. To begin, staff must review an EMR manually. This takes considerable time and effort as the patient’s demographics, comorbidities, inclusion criteria, and a sea of other information needs to be identified. Once the information is gathered, decision-making must commence, which in itself is an entirely tiresome process, involving the prolongation of patient’s stay as well when said patient is interviewed regarding enrollment. Needless to say, many eligible candidates are discouraged by this type of approach and are disinterested. Financial burden due to longer visits also factors in, for the institution as well as the individual. The role of machine learning here is to automate the input (EHR data) and to yield an output (decision on patient eligibility). Current barriers to advancement in these technologies are numerous. However, the two pressing ones now are: (a) data dispersed in different sources; such as different departments and institutions, (b) absence of clear guidelines relating to how data are processed prior to being analyzed by an AI system and (c) lack of adequate descriptions detailing how using AI systems affects healthcare outcomes.^27^

Fortunately, solutions to these issues are being pursued with CONSORT-AI and SPIRIT-AI as they continue to establish guidelines to prioritize efficacy in healthcare with real-time implementation.^27^ These guidelines should clearly outline the methodology that includes: how AI was incorporated, how AI guides clinical decisions, and the discussions on who will use AI to make decisions and what these decisions are. Additionally, detailing standardized guidelines for AI intervention will hopefully propel the field in the right direction. AI research is definitely motivated to enter the market, which is naturally going to be subject to “AI-chasm.” There exists adaptability issues of AI using data that do not translate to the real world. For instance, an algorithm may use retrospective data, or data drawn from outside the healthcare setting. This creates barriers to translation into the real world.^28^ Thus, we enter a chasm of AI systems having reasonable solutions, but struggling to make meaningful impacts in healthcare.

In conclusion, AI systems are showing promising results. However, there exists a clear lack of evidence demonstrating applicability to real-world healthcare. Many AI systems as mentioned above accrued larger patient cohorts under quicker recruitment times when emulating a recruitment process. More promising study designs in the future would hopefully include real clinical trial recruitment processes using AI systems in order to analyze their efficacy. It remains a strong promise that AI intervention, along with guidelines to facilitate its growth, will bolster the impact of clinical trials by increasing number of participants and reducing time to recruitment. This will allow for stronger clinical trials with higher accrual rates, idealized patient composition, and more motivation to pursue clinical trials when the recruitment process is streamlined and automated.
